# Psychosocial and Clinical Correlates of Somatic Symptom Disorder in Patients With and Without Somatic Comorbidities: Cross-Sectional Findings From the SOMA.SSD Study

**DOI:** 10.1097/PSY.0000000000001483

**Published:** 2026-04-14

**Authors:** Henrike Wittenbecher, Stefanie Hahn, Yvonne Nestoriuc, Kristina Blankenburg, André Strahl, Franz Pauls, Kerstin Maehder, Bernd Löwe, Anne Toussaint

**Affiliations:** Department of Psychosomatic Medicine and Psychotherapy, University Medical Center Hamburg-Eppendorf, Hamburg, Germany (Wittenbecher, Blankenburg, Strahl, Maehder, Löwe, Toussaint); Institute of Systems Neuroscience, University Medical Center Hamburg-Eppendorf, Hamburg, Germany (Nestoriuc); Clinical Psychology and Psychotherapy, Helmut-Schmidt-University/University of the Federal Armed Forces, Hamburg, Germany (Hahn, Nestoriuc, Pauls)

**Keywords:** persistent somatic symptoms, somatic symptom disorder, medical comorbidity, cumulative illness rating scale, diagnostic validity, health anxiety, **CI** = Comorbidity Index, **CIRS** = Cumulative Illness Rating Scale, **DSM-5** = Diagnostic and Statistical Manual of Mental Disorders, 5th Edition, **DSM-IV** = Diagnostic and Statistical Manual of Mental Disorders, 4th Edition, **GAD-7** = Generalized Anxiety Disorder Scale-7, **HCU** = health care utilization, **HrQoL** = health-related quality of life, **ICD-10** = International Statistical Classification of Diseases and Related Health Problems, 10th Revision, **ISRCTN** = International Standard Randomised Controlled Trial Number, **MCS** = Mental Component Scale, **PCS** = Physical Component Scale, **PHQ-15** = Patient Health Questionnaire-15, **PHQ-9** = Patient Health Questionnaire-9, **PSS-10** = Perceived Stress Scale-10, **SCID-5** = Structured Clinical Interview for DSM-5, **SCQ-D** = Self-Administered Comorbidity Questionnaire, **SF-12** = Short Form-12, **SPSS** = Statistical Package for the Social Sciences, **SSAS** = Somatosensory Amplification Scale, **SSD** = somatic symptom disorder, **SSD-12** = Somatic Symptom Disorder-B-Criteria Scale-12, **WI-7** = Whiteley Index-7

## Abstract

**Objective:**

Somatic symptom disorder (SSD) is characterized by persistent physical symptoms causing significant distress. Unlike earlier criteria, SSD does not require the absence of other medical conditions, resulting in a heterogeneous patient population. Few studies have systematically compared SSD patients with and without somatic comorbidities. This study examined the frequency of somatic comorbidities in SSD and their associations with psychosocial characteristics and disorder severity.

**Methods:**

Cross-sectional data from the SOMA.SSD study were analyzed. SSD was diagnosed through the Structured Clinical Interview for the Diagnostic and Statistical Manual of Mental Disorders, 5th edition (DSM-5). Somatic comorbidity was assessed with the physician-rated Cumulative Illness Rating Scale (CIRS). Patients with and without somatic comorbidity were compared on psychosocial and clinical variables. Ordinal logistic regression identified factors associated with SSD severity.

**Results:**

A total of 241 SSD patients were included from a psychosomatic outpatient clinic (mean age: 44.5 y, SD 13.7; 66.8% female). SSD severity was mild in 35.7%, moderate in 38.6%, and severe in 25.7%. Mean CIRS score was 4.4 (SD: 3.18; range: 0 to 52), with 61.0% of patients having at least one somatic comorbidity. Patients with somatic comorbidity were older and reported lower physical but higher mental health-related quality of life. Higher health anxiety was associated with greater SSD severity (*β*=0.287, *p*<.001), whereas somatic comorbidity was not linked to severity or psychosocial measures.

**Conclusions:**

Somatic comorbidities were common in SSD but did not influence severity or psychosocial profile, supporting the validity of the diagnosis independent of physical disease. Health anxiety emerged as a key correlate of severity, highlighting its relevance as a treatment target.

## INTRODUCTION

The American Psychiatric Association introduced *Somatic Symptom Disorder* (SSD) as a new diagnosis in the Diagnostic and Statistical Manual of Mental Disorders, 5th edition (DSM-5) in 2013. Its diagnostic criteria include the presence of at least one distressing somatic symptom (A-criterion), accompanied by excessive thoughts, feelings, or behaviors (B-criteria) for at least 6 months (C-criterion).^1^ Correspondingly, the 11th edition of the International Classification of Diseases (ICD-11) introduced the diagnosis of bodily distress disorder, defined by the persistence of (typically multiple) distressing bodily symptoms accompanied by excessive attention toward these symptoms.^2^ This focus on the subjective burden aligns with the broader transdiagnostic framework of persistent somatic symptoms, which integrates distressing physical symptoms across various somatic conditions, functional syndromes, and mental disorders, rather than viewing them in isolation.^3^ A related approach, Bodily Distress Syndrome (BDS), emphasizes empirically derived clusters of functional somatic symptoms across and within organ systems, acknowledging that symptom patterns may involve single or multiple organ domains.^4^

On the basis of self-report questionnaires, prevalence estimates for SSD in the general population vary between 6.4% and 17.4%.^5^ Unlike earlier concepts, including the notion of medically unexplained symptoms (MUS),^3^ SSD no longer requires symptoms to be medically unexplained and can now be diagnosed alongside existing somatic conditions.

This paradigm shift—compared with the previous concept of somatoform disorders as defined by the Diagnostic and Statistical Manual of Mental Disorders, 4th edition (DSM-IV)^6^ and ICD-10^7^—has led to substantial heterogeneity among patients within a single diagnostic category and raises the question of whether uniform diagnostic criteria are appropriate for individuals with varying degrees of somatic comorbidity.^8^ Critics fear potential overinclusiveness of medically ill patients on the one hand and the risk of mislabeling patients without a somatic diagnosis as mentally ill on the other hand.^9^ Proponents, however, see an opportunity to offer beneficial psychological support to more patients in need.^5^ One proposed solution is the introduction of diagnostic subtypes in order to distinguish SSD patients with and without somatic comorbidity.^8^ In this context, the assessment and understanding of somatic comorbidity and its role in SSD have gained increasing importance.^10^

Previous research has demonstrated that psychological factors reflected by the SSD B-criteria are associated with somatic comorbidity in both the general population^11^ and primary care.^12^ Individuals meeting SSD B-criteria had higher rates of physical illnesses and increased health care utilization (HCU) in the general population.^11^ In primary care, physical comorbidities correlated with higher scores on the SSD B-criteria scale.^12^ A population-based study showed that the risk of SSD increased with the number of somatic comorbidities in patients with a major medical disorder.^13^ In contrast, a hospital outpatient study reported no association between medical conditions and SSD diagnosis or related psychological measures, though somatic comorbidity was assessed only by self-report.^14^ Similarly, no link was found between the diagnosis of SSD and disease-specific parameters in patients with breast cancer.^15^ A cohort study of patients with SSD found no significant relationship between somatic comorbidity and SSD severity. However, results were limited by a relatively small sample size and the use of an unstructured SSD assessment.^16^ Overall, current research on the role of somatic comorbidity in SSD largely relies on self-report measures, showing inconsistent findings, and its relationship to specific characteristics of SSD remains unclear.^5^ To gain a more profound understanding of SSD, it is essential to explore whether the presence of somatic comorbidity is associated with the symptom burden and clinical presentation of SSD.

Patients with SSD frequently experience reduced health-related quality of life (HrQoL) compared with the general population^5^ and tend to show higher HCU rates than non-SSD patients in general hospital care^14^ or the general population.^5^ Across several studies, greater functional impairment, higher HCU, and poorer quality of life have been consistently associated with increasing SSD severity.^5^ Patients also report higher levels of depressivity, alexithymia scores, general anxiety, and health anxiety symptoms.^17^

This study (1) characterized somatic comorbidity in patients diagnosed with SSD, (2) examined the correlation between physician-rated somatic comorbidity and patient self-reported somatic comorbidity, (3) aimed to clarify whether somatic comorbidity is cross-sectionally associated with SSD severity, and (4) compared patients with and without somatic comorbidity regarding relevant psychological variables as well as clinically relevant outcomes such as somatic symptom severity, HCU, and HrQoL.

## METHODS

### Transparency and Openness

The present study was part of the research project “SOMA.SSD—Modifiable factors for somatic symptom persistence in patients with somatic symptom disorder,” a prospective single-center cohort study investigating risk factors for somatic symptom persistence in patients with SSD.^10^ SOMA.SSD is one of the projects of the Research Unit 5211 (FOR 5211), “Persistent SOMAtic Symptoms ACROSS Diseases: From Risk Factors to Modification (SOMACROSS),”^18^ funded by the German Research Foundation [Deutsche Forschungsgemeinschaft (DFG)] (grant numbers NE 1635/3-1 and TO 908/2-1).^10^

The study design and procedures were approved by the Ethics Committee of the Hamburg Medical Association, Germany (reference number: 2020-10197-BO-ff, January 25, 2021). Detailed information on size determination, study procedures, and measures is provided in the study protocol.^7^ The study was preregistered at ISRCTN: https://www.isrctn.com/ISRCTN36251388.

Due to privacy considerations, study data and analysis code are available upon reasonable request from the corresponding author.

### Participants and Procedure

Patients were recruited between May 2022 and July 2023 at the psychosomatic outpatient clinic of the Department of Psychosomatic Medicine and Psychotherapy of the University Medical Center Hamburg-Eppendorf, Hamburg, Germany. Patients were prescreened using the Patient Health Questionnaire-15 (PHQ-15) and the Somatic Symptom Disorder-B-Criteria Scale-12 (SSD-12) as part of standard routine care. Patients who scored above the cutoffs (PHQ-15 scores ≥5 and SSD-12 scores ≥20) were contacted and informed about the study. Trained members of the study team conducted telephone interviews to confirm the diagnosis of SSD and assess its severity. Further inclusion criteria were age 18 years or older, permission to review medical records, sufficient oral and written German language proficiency, and written informed consent. Exclusion criteria were serious illnesses requiring immediate intervention, florid psychosis or substance use disorder, acute suicidality, or simultaneous participation in other clinical trials.

As part of the SOMA.SSD project, each participant attended 3 study appointments. All analyses of the present study are based exclusively on the data collected during the baseline assessment that consisted of questionnaires, medical anamnesis, physical examination, and review of medical records.

### Instruments

#### Self-Report Questionnaires

Participants completed the questionnaire core set of the RU SOMACROSS (for a detailed overview, see the study protocol),^18^ along with study-specific questionnaires. The following instruments, relevant to the current research questions, were included in the present study.

#### Patient Health Questionnaire-15

The PHQ-15 was used to assess somatic symptom severity within the last 4 weeks, with symptoms rated from 0 (“not bothered at all”) to 2 (“bothered a lot”) and a total score of 0 to 30. The internal reliability of the scale (Cronbach *α*=0.80)^19^ and validity in psychosomatic samples have been shown.^20^

#### Somatic Symptom Disorder-B-Criteria Scale-12

The SSD-12 assesses the psychological distress related to somatic symptoms across 12 items measuring the 3 DSM-5 B-criteria, rated from 0 (“never”) to 4 (“very often”). The total score ranges from 0 to 48, with high reliability (Cronbach *α*=0.94) and validity.^21^

#### Patient Health Questionnaire-9

As a frequent psychological comorbidity of SSD associated with poorer functioning,^22^ depression severity was assessed using the Patient Health Questionnaire-9 (PHQ-9). It measures the severity of depressive symptoms over the past 2 weeks. It uses 9 items scored from 0 (“not at all”) to 3 (“nearly every day”), yielding a total sum score from 0 to 27.^23^ Validity and reliability (Cronbach *α*=0.86 to 0.89) were demonstrated.^23,24^

#### Whiteley Index-7

Health anxiety was measured using the Whiteley Index-7 (WI-7). It consists of 7 dichotomous (“yes”/“no”) questions and provides a total sum score ranging from 0 to 7. Validity and reliability (Cronbach *α*=0.83) was demonstrated for the WI-7.^25,26^

#### Generalized Anxiety Disorder Scale-7

Generalized anxiety severity within the last 2 weeks was assessed using the Generalized Anxiety Disorder Scale-7 (GAD-7),^27^ which rates the 7 DSM-IV GAD symptoms from 0 (“not at all”) to 3 (“nearly every day”) (total score 0 to 21). The scale shows high reliability (Cronbach *α*=0.92) and criterion validity.^28^

#### Perceived Stress Scale

Perceived stress during the past month was assessed using the Perceived Stress Scale-10 (PSS-10). Ten items are rated from 0 (“never”) to 4 (“very often”) with a sum score from 0 to 40. The PSS-10 has demonstrated sufficient reliability (Cronbach *α*=0.77) and validity.^29^

#### Somatosensory Amplification Scale

The Somatosensory Amplification Scale (SSAS) requires respondents to rate 10 statements about somatization from 1 (“not at all true”) to 5 (“extremely true”). The total sum score ranges from 0 to 50. Reliability (Cronbach *α*=0.82) and validity have been confirmed.^30^

#### Self-Administered Comorbidity Questionnaire

The Self-Administered Comorbidity Questionnaire (SCQ-D) was used as a self-report to gather information on the presence of 13 health problems (eg, “heart disease” and “cancer”), their treatment, and their impact on daily life. Each category can be answered with “yes” or “no.” The item “depression” was excluded in order to focus exclusively on somatic health problems. Total sum scores range from 0 to 36.^31^ Internal consistency is good (Cronbach *α*>0.78).^32^

#### Short Form-12

The Short Form Health Survey-12 (SF-12) was used to assess HrQoL. The concluding Physical Component Scale (PCS) and Mental Component Scale (MCS) can each reach a total scale score ranging between 0 and 100.^33^ Good validity and acceptable reliability could be shown for both scales, with Cronbach *α*=0.77 for the PCS and Cronbach *α*=0.80 for the MCS.^34^

#### Health Care Utilization

Patients were asked with 2 open items about the total number of medical consultations they had attended within the last 4 weeks and 6 months.

### Rater-Administered Interviews

#### Diagnosis of SSD

The diagnosis of SSD was made using the research version of the German SSD-section of the Structured Clinical Interview for DSM-5 (SCID-5).^20^ SSD severity was rated as mild (1 criterion B symptom), moderate (2 or more criterion B symptoms), or severe (2 or more criterion B symptoms accompanied by either multiple somatic complaints or one very severe somatic complaint).

#### Assessment of Somatic Comorbidities

Somatic comorbidity was assessed by a trained physician using the Cumulative Illness Rating Scale (CIRS), an instrument for measuring multimorbidity and comorbidity in patients by evaluating the severity of impairment in different organ systems.^35,36^ In the present study, the 14-organ system version by Miller et al^35^ was used, excluding the section on psychiatric illness in order to focus exclusively on somatic comorbidity. Included organ systems for evaluation were cardiological; vascular; hematological; respiratory; ears, eyes, nose, and throat; upper gastrointestinal; lower gastrointestinal; hepatic and pancreatic; renal; genitourinary; musculoskeletal, tegumental; neurological, endocrine, metabolic, and breast. The extent of impairment in each organ system is rated on a scale from 0 to 4 (no/mild/moderate/severe/extremely severe impairment). Ratings were thereby based on the anamnestic interviews, the physical examination, and review of patients’ medical records. The CIRS total sum score was based on 13 individual scores, thus ranging from 0 to 52. The CIRS comorbidity index (CI) represents the number of organ systems rated 2 or higher. For the purpose of group comparisons, patients were categorized into 2 categories based on their CI: those with a CI of 0 (indicating that no organ system was rated as moderately impaired or higher) were classified as “without somatic comorbidity,” while those with a CI of 1 or higher (indicating relevant impairment in at least one organ system) were classified as “with somatic comorbidity.” This binary split was selected to provide a clear distinction between patients with and without somatic comorbidity and to maintain adequate group sizes for statistical analyses.

### Statistical Analysis

All statistical analyses were conducted using IBM SPSS Statistics, Version 29.^37^ Descriptive statistics were calculated to provide relevant information about sociodemographic characteristics, psychological variables, SSD severity, and the presence of somatic comorbidities across the entire sample. Correlational analyses were conducted to analyze associations between somatic comorbidity, psychological variables, and clinically relevant outcomes (ie, HrQoL, HCU), as well as to examine the concordance between physician-rated and self-reported somatic comorbidity. In order to identify any potential group differences between patients with and without somatic comorbidity, independent-samples *t* tests were used for continuous variables, while χ^2^ tests were used for categorical variables. Effect sizes were reported, including Cohen *d* (*t* tests) and Cramer V (χ^2^ tests). Group comparisons in Table 1 are exploratory, and *P*-values are reported without adjustment for multiple testing. To explore the association of somatic comorbidity and other factors with the severity of SSD, categorized as mild, moderate, or severe, an ordinal logistic regression analysis was conducted. Psychosocial variables for the regression analysis were selected a priori based on established theoretical models of SSD, representing maladaptive illness-related cognitions (illness anxiety and somatosensory amplification), negative affectivity (depression and anxiety), and stress-related vulnerability (perceived stress), which have consistently been shown to be associated with symptom persistence and severity.^10^ Age and sex were included as standard demographic covariates. Somatic comorbidity was entered to account for objective somatic health burden. Other sociodemographic and outcome-related variables were excluded to preserve model parsimony and reduce the risk of overfitting. Nagelkerke *R*² was reported to estimate the proportion of variance explained by the model, facilitating interpretation of the regression results. Standardized regression coefficients (β) were used as measures of effect size. To account for multiple testing in the ordinal logistic regression analysis, the Bonferroni correction was applied. On the basis of 8 regression coefficients included in the model, the adjusted significance threshold was set at *p*<.00625. This conservative approach was chosen to control the family-wise error rate, although it may increase the risk of type II error. Accordingly, only results below this threshold were considered statistically significant.

**TABLE 1:** Demographic and Clinical Characteristics of the SOMA.SSD Sample (*n*=241)

Variable	Total sample (*N*=241)	With somatic comorbidity (*n*=147)	Without somatic comorbidity (*n*=94)	*t*/χ^2^	*df*	Effect size (|*d*|/*V*)	*p*
Age	44.5 (13.7)	47.4 (14)	39.8 (12)	−4.51	218	0.58	**<.001**
Sex							
Female	161 (66.8%)	101 (68.7%)	60 (63.8%)	0.615	1	0.05	.433
Education							
>10 y	162 (76.2%)	95 (64.6%)	67 (71.3%)	1.151	1	0.07	.283
Employment							
Full-time employed	94 (39%)	56 (38.1%)	38 (40.4%)	0.131	1	0.02	.718
Work incapacity							
Yes (<6 wk)	7 (2.9%)	4 (2.7%)	3 (3.2%)	0.564	2	0.05	.754
Yes (>6 wk)	102 (42.3%)	65 (44.2%)	37 (39.4%)				
Migration background							
Yes	49 (20.4%)	27 (18.5%)	22 (23.4%)	0.849	1	0.06	.357
SSD severity							
Mild	86 (35.7%)	56 (38.1%)	30 (31.9%)	1.718	2	0.08	.424
Moderate	93 (38.6%)	52 (35.4%)	41 (43.6%)				
Severe	62 (25.7%)	39 (26.5%)	23 (24.5%)				
Total no. health care visits							
Last 4 wk	3.0 (3.0)	3.2 (3.1)	2.6 (2.7)	−1.641	239	0.22	.102
Last 6 mo	12.4 (10.5)	12.4 (9.6)	12.4 (11.7)	−0.007	238	0.00	.994
Health-related quality of life (SF-12)							
Physical component (0-100)	37.3 (10.2)	35.4 (9.7)	40.2 (10.1)	3.651	237	0.48	**<.001**
Mental component (0-100)	41.2 (7.5)	42.1 (7.6)	39.8 (7.2)	−2.233	237	0.30	**.026**
Somatic comorbidity self-report (SCQ-D) (0-36)	5.6 (4.0)	6.7 (4.3)	4 (3.0)	−5.820	237.3	0.71	**<.001**
Somatic symptom burden (PHQ-15) (0-30)	12.3 (5.1)	12.7 (5.1)	11.6 (4.9)	−1.555	238	0.21	.121
Somatic Symptom Disorder B-Criteria (SSD-12) (0-48)	28.2 (9.0)	28.5 (8.6)	27.8 (9.6)	−0.590	237	0.08	.556
Depression severity (PHQ-9) (0-27)	12.1 (5.5)	12.2 (5.4)	11.9 (5.6)	−0.505	237	0.07	.556
Anxiety severity (GAD-7) (0-21)	10.7 (5.3)	10.8 (5.3)	10.6 (5.2)	−0.359	238	0.05	.720
Illness anxiety (WI-7) (0-7)	4.4 (1.8)	4.5 (1.7)	4.1 (2.0)	−1.754	169.2	0.24	.082
Perceived stress (PSS-10) (0-40)	23.4 (6.7)	23.5 (6.7)	23.3 (6.9)	−0.270	237	0.04	.787
Somatosensory amplification (SSAS) (10-50)	15.8 (7.3)	16.1 (7.8)	15.2 (6.4)	−1.055	222.6	0.13	.293

Data are presented as M (SD) or *n* (%). χ^2^ tests were used for categorical variables and *t* tests for continuous variables.

SSD = Somatic Symptom Disorder; SF-12 = Short Form Health Survey; SCQ-D = Self-administered Comorbidity Questionnaire (depression excluded); PHQ-15 = Patient Health Questionnaire-15; SSD-12 = Somatic Symptom Disorder B-criteria Scale; PHQ-9 = Patient Health Questionnaire-9; GAD-7 = Generalized Anxiety Disorder Scale-7; WI-7 = Whiteley Index-7; PSS-10 = Perceived Stress Scale-10; SSAS = Somatosensory Amplification Scale.

## RESULTS

### Study Sample

A total of *N*=1594 patients with an initial consultation at the psychosomatic outpatient clinic between May 2022 and July 2023 were deemed eligible for screening at baseline. SSD diagnosis could be confirmed in 271 patients through SCID interview. Since 30 patients objected to study participation, a total of *N*=241 participated in the baseline assessment. The flow of participants is depicted in Figure S1, Supplemental Digital Content, http://links.lww.com/PSYMED/B168.

The mean age of the total sample was 44.5 years (SD=13.7), and 66.8% reported female sex (0% diverse). Regarding SSD severity, 35.7% of participants met the criteria for a mild SSD, 38.6% for a moderate SSD, and 25.7% for a severe SSD. A total of 76.2% of the cohort had more than 10 years of formal education, and 39.0% were full-time employed. Regarding work incapacity, 2.9% reported being unable to work for <6 weeks, while 42.3% reported an incapacity lasting 6 weeks or longer. Migration background was indicated if the patient or at least one parent was born outside of Germany. On the basis of this definition, 20.4% of participants were classified as having a migration background. For a detailed overview, see Table 1.

### Somatic Comorbidity

The mean CIRS total score was 4.4 (SD=3.17), with a range of scores from 0 to 16. As indicated by a CIRS total score ≥1, the most frequently affected and at least mildly impaired organ systems were the musculoskeletal system (49.4%), the vascular system (35.3%), and the ears, eyes, nose, and throat system (34%). The most severe impairments were recorded in the cardiological system (3.7% with severe impairment). No organ system was rated as extremely severely impaired, since patients with serious illnesses requiring immediate intervention had been excluded beforehand. The 5 most prevalent underlying diagnoses were arterial hypertension (21.2%), bronchial asthma (11.6%), migraine (10.4%), herniated disc (9.1%), and hypothyroidism (7.9%).

**FIGURE 1:**
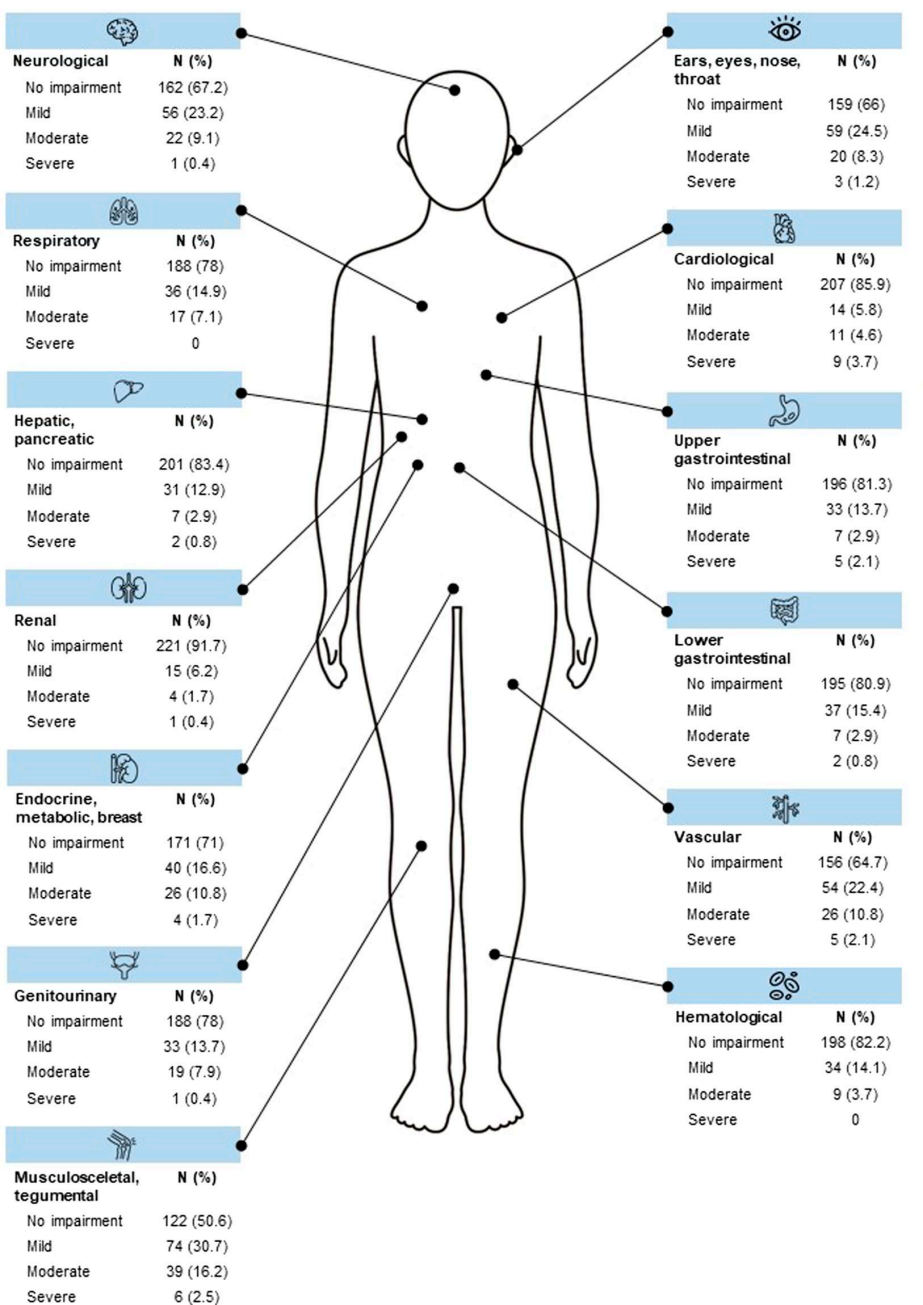
Organ system-specific scores of the Cumulative Illness Rating Scale (CIRS) in the *n*=241 patients of the SOMA.SSD study. *Color image is available online only at the journal’s website*.

A total of *n*=94 patients (39%) with a CI of 0 were categorized as “without somatic comorbidity.” Consequently, *n*=147 patients (61%) were assigned a CI of 1 or higher and were accordingly categorized as “with somatic comorbidity.” In this group, *n*=76 patients (31.5%) achieved a CI of 1, *n*=47 patients (19.5%) achieved a CI of 2, *n*=15 patients (6.2%) achieved a CI of 3, *n*=4 patients (2.9%) achieved a CI of 4, and *n*=5 patients (0.8%) achieved a CI of 5. For a detailed overview of the CIRS scores, see Fig. 1.

### Correlation of Clinician-Assessed and Self-Reported Somatic Comorbidity

Somatic comorbidity as measured by the CIRS showed a significant positive, strong correlation (according to Cohen^38^) with the self-reported somatic comorbidity (SCQ-D) (*r*=0.61, *p*<.001). Higher CIRS total scores were thus found to be significantly associated with higher scores in the SCQ-D.

### Correlation of Somatic Comorbidity With Clinical and Psychological Variables

Higher levels of somatic comorbidity, as indicated by higher CIRS total scores, were found to be significantly associated with higher levels of somatic symptom severity (PHQ-15; *r*=0.24, *p*<.001), reduced HRQoL, physical component (SF-12 PCS; *r*=−0.35, *p*<.001) and greater health anxiety (WI-7; *r*=0.20, *p*<.001). For all correlations of CIRS ratings with clinical and psychological variables, see Table S1, Supplemental Digital Content, http://links.lww.com/PSYMED/B169.

### Association of Somatic Comorbidity and SSD Severity

The ordinal logistic regression model with age, sex, illness anxiety, depression severity, anxiety severity, perceived stress, somatosensory amplification, and somatic comorbidity as predictor variables and SSD severity as the criterion variable was found to be statistically significant (*χ* ^2^(8)=21.028, *p*=.007), indicating that at least one included predictor variable was significantly associated with SSD severity. There was no significant effect of somatic comorbidity on SSD severity (*β*=−0.059, *p*=.608). Among the 8 psychosocial variables included as predictor variables in the model, health anxiety emerged as the only one significantly affecting SSD severity (β=0.287, *p*<.001). The model explained ∼9.5% of the variance in SSD severity, as indicated by a Nagelkerke *R* ^2^ of 0.095 (Table 2).

**TABLE 2:** Ordinal Logistic Regression for Association With Somatic Symptom Disorder Severity (*n*=241)

			95% CI				
Variable	*β*	SE *β*	Lower	Upper	Wald χ^2^	*df*	*p*
Age	0.005	0.009	−0.013	0.024	0.314	1	.576
Sex	−0.250	0.272	−0.783	0.283	0.846	1	.358
Illness anxiety (WI-7)	0.287	0.079	0.133	0.441	13.296	1	**<.001**
Depression severity (PHQ-9)	−0.001	0.037	−0.072	0.071	0	1	.988
Anxiety severity (GAD-7)	−0.001	0.041	−0.082	0.082	0	1	.983
Perceived stress (PSS-10)	−0.012	0.028	−0.067	0.042	0.191	1	.662
Somatosensory amplification (SSAS)	0.018	0.022	−0.024	0.061	0.719	1	.396
Somatic comorbidity (CIRS CI)	−0.059	0.115	−0.284	0.166	0.263	1	.166

Bonferroni-adjusted significance threshold was set at *p*<.00625.

WI-7 = Whiteley Index-7; PHQ-9 = Patient Health Questionnaire-9; GAD-7 = Generalized Anxiety Disorder-7; PSS-10 = Perceived Stress Scale-10; SSAS = Somatosensory Amplification Scale; CIRS = Cumulative Illness Rating Scale; CI = Comorbidity Index.

### Group Comparison of Sociodemographic Characteristics

The group with somatic comorbidity, defined by a comorbidity index of 1 or higher, comprised a total of *n*=147 patients [68.7% female, mean age 47.4 y (SD=13.9)]. The group without somatic comorbidity consisted of *n*=94 patients [63.8% female, mean age 39.8 y (SD=12.0)]. Patients with somatic comorbidity were significantly older than those without (*t*(218)=−4.509; *p*<.001, *d*=0.576). Both groups did not differ significantly regarding educational background, employment situation, work incapacity, migration background, or SSD severity. Sociodemographic characteristics of the total cohort, as well as group differences, are presented in Table 1.

### Group Comparisons for Clinically Relevant Outcomes and Psychological Variables

Patients with SSD and somatic comorbidity reported a significantly lower HRQoL regarding the physical component (*t*(237)=3.651; *p*<.001, *d*=0.484), while achieving significantly higher scores on the mental component (*t*(237)=3.651; *p*=.026, *d*=−0.296) compared with patients without somatic comorbidity. Patients with somatic comorbidity also showed significantly higher levels of self-reported comorbidity (*t*(237.3)=−5.820; *p*<.001, *d*=0.711). However, no group differences were found for psychological variables and HCU.

## DISCUSSION

The study investigated the prevalence of somatic comorbidity in a sample of patients with SSD and its association with sociodemographic and psychological characteristics, HrQoL, HCU, and SSD severity within a psychosomatic outpatient setting. On the basis of predefined criteria, more than 60% of patients were classified as having a somatic comorbidity after comprehensive consultation and structured diagnostic assessment by a clinician. Patients with somatic comorbidity reported significantly lower levels of health-related quality of life; however, no significant group differences were found in the other examined variables. Moreover, no significant effect of somatic comorbidity could be found on SSD severity.

The role of somatic comorbidity in patients with SSD has become particularly relevant as the change in the diagnostic approach introduced in DSM-5 has sparked widespread discussion and raised concerns about potential overdiagnosis in patients with medical conditions.^9^ To date, only a few studies have systematically examined the impact of somatic comorbidity on the diagnostic meaning of SSD, and existing evidence is limited by either the use of self-reported comorbidity data^14,16^ or the absence of a standardized clinical diagnosis of SSD.^11,12,16^ In the present study, SSD diagnosis was based on the structured clinical interview SCID-5, and somatic comorbidity was assessed by trained clinicians (CIRS).

Results of the present study demonstrated that 61% of patients with SSD had at least one relevant somatic comorbidity. The most frequently affected organ systems were the musculoskeletal and vascular systems. These findings align with results from a previous study in an Australian online clinical setting in which patients with SSD most frequently reported asthma, circulatory conditions, gout, rheumatism, and arthritis as somatic conditions.^39^

In the present study, a significant difference between the patients with and without somatic comorbidity was found regarding age and health-related quality of life. On average, patients with somatic comorbidity were older than those without somatic comorbidity, which may reflect the association of older age and a higher risk of multimorbidity, as shown in a cross-sectional study in Germany.^40^

Patients with somatic comorbidity also reported a lower HrQoL in the physical domain, while exhibiting a higher HrQoL in the mental domain compared with those without somatic comorbidity. Explanatory approaches for the reduced physical HrQoL in patients with medical illnesses typically include limitations in daily functioning and medication use,^41^ reduced physical activity,^42^ and treatment-related side effects.^43,44^ By contrast, mental HrQoL was found to be lower in patients without somatic comorbidity in the present study. This finding suggests that somatic symptom severity on its own might not be the primary factor affecting the HrQoL in patients with SSD. It is more likely that other factors may also play a crucial role. Previous studies have identified a meaningful association between comorbid depression and reduced overall HrQoL in patients with SSD^22^ and those with medical illnesses.^44^ However, in the present sample, the 2 groups did not differ in self-reported depression severity or other psychological variables. It should be noted that comorbid psychiatric diagnoses were not analyzed in the present study, and only self-report measures were used. In the present study sample, both groups reported high levels of somatic symptom severity, but only one group had a diagnosed medical condition that might explain their somatic symptoms. The uncertainty associated with not having a clear medical explanation for the individually experienced symptoms may thus negatively affect overall mental well-being in various ways.

A meta-analysis by McAndrew et al^45^ applied Leventhal's common-sense model of self-regulation to better understand health outcomes in patients with medically unexplained symptoms. According to the model, individuals develop cognitive illness representations that shape their emotional responses, coping strategies, and health outcomes.^46^ The meta-analysis revealed that threat-related representations and attributing symptoms to psychological rather than somatic causes were linked to poorer health outcomes, such as reduced general quality of life. In contrast, patients with a somatic diagnosis (ie, somatic comorbidity in the present study sample) may have developed a sense of control and coherence regarding their symptoms, which is considered a protective representation and may contribute to a better quality of life.^45^ However, variables such as illness perception and coping were not analyzed in this study, so more precise conclusions regarding the 2 groups cannot be drawn at this point. Moreover, patients with so-called “medically unexplained symptoms” face several barriers when it comes to access to health care services, such as negative attitudes and limited knowledge of symptom-related disorders.^47^ Stigmatization, as frequently experienced by patients with persistent somatic symptoms during medical consultations, may represent an additional burden.^48^

Altogether, the present study sample featured elevated levels of somatic symptom severity, psychological distress related to somatic symptoms, depression severity, anxiety severity, and health anxiety when compared with the German general population.^11,24,49–51^ This aligns with findings from a recent meta-analysis that found higher levels of depressive symptoms, symptoms of anxiety, and health anxiety in patients with SSD compared with healthy controls.^17^ Notably, patients in the present sample also reported more health care visits within 6 months than the German general population did over an entire year.^52^ Previous studies have similarly reported an association between higher SSD-12 scores and increased HCU in both general population samples^11^ and a psychosomatic outpatient setting.^21^ However, no meaningful cross-sectional differences were found in the named variables between patients with and without somatic comorbidity. The psychological burden and somatic symptom severity in patients with SSD, therefore, do not appear to be substantially associated with the presence of somatic comorbidity at a cross-sectional level. This does not preclude the possibility that smaller effects or specific mechanisms (eg, differences in illness perceptions, coping strategies, or longitudinal symptom trajectories) exist but were not captured in the present study.

Similar conclusions were drawn in a study that examined the risk of SSD in a general population sample with at least one self-reported major medical disease. Individuals with a major medical disease did not report an excessive somatic symptom burden (measured by the Somatic Symptom Scale-8) or significant psychological distress related to these symptoms (measured by the SSD-12). Moreover, people with a major medical disease were not at increased risk for SSD compared with the general population.^13^ In contrast, Kop et al^11^ found that the presence of a medical disorder was associated with increased SSD-12 scores in a Dutch general population sample, with high values in patients with a major medical disease that requires monitoring in a specialized health care setting. Notably, even patients with medical conditions that are commonly treated in primary care exhibited elevated SSD-12 scores, indicating that even mild conditions may represent a relevant burden for patients. In the same study, the association of SSD-12 scores and HCU remained independent of the presence of medical comorbidities. However, it should be noted that those results are based on a general population sample with no formal diagnosis of SSD.^11^ Increased HCU was also independent of the presence of medical comorbidities in a large cohort of patients with somatic symptoms and related disorders.^53^

Results of the present study highlight the capacity of the reconceptualized diagnostic criteria of SSD to identify patients who are impaired by physical symptoms, irrespective of the presence of any somatic comorbidity. The findings support the assumption that the diagnosis can be applied to patients with and without somatic comorbidity, and that the identified patients are truly in need of psychological treatment. Consequently, SSD screening and treatment pathways should not exclude medically ill patients, as their psychological burden and SSD severity appear comparable. Given the robust association between health anxiety and SSD severity, health anxiety warrants explicit assessment and targeted treatment, for example, within cognitive-behavioral therapy protocols focusing on symptom misinterpretation and safety behaviors.

The results of the ordinal logistic regression failed to indicate any associations between somatic comorbidity and SSD severity. Furthermore, there were no significant differences in SSD severity between patients with and without somatic comorbidity. On the one hand, these results support findings from a prior study suggesting that the severity of SSD is independent of the presence of somatic comorbidity.^16^ Similarly, another study found that in patients with SSD, high complexity (as assessed by the INTERMED, a tool evaluating patient needs) was not associated with a greater number of somatic symptoms.^54^ On the other hand, somatic comorbidity was found to be meaningfully associated with an increased risk of SSD in patients with a major medical disorder; that is, a higher number of somatic comorbidities was linked to a higher risk of SSD.^13^ Although somatic comorbidity did not emerge as a significant predictor in this model (*p*=.608), this finding should be interpreted with caution. Given the modest sample size and the overall model variance explained (*R* ^2^=0.095), the study may have been underpowered to detect smaller, yet potentially clinically relevant, associations (type II error). As the present regression analysis indicated, health anxiety was the only factor that was significantly affecting SSD severity. For each one-point increase on the WI-7 (health anxiety), the odds of being in a higher SSD severity category increase by about 33%, independent of other variables. Although the overall regression model was statistically significant, the proportion of explained variance was rather small, with only 9.5% of SSD severity accounted for by the included predictors. This underscores the complexity and multifactorial nature of SSD and suggests that many relevant influences remain unmeasured. Future research must move beyond single predictors and systematically examine a broader range of psychosocial and biological factors, including potential trait and state mechanisms, to identify key determinants of SSD severity and inform targeted interventions.

Nevertheless, higher illness anxiety levels were previously found in patients with severe SSD compared with those with a mild or moderate SSD.^16^ A recent systematic review underlines the crucial role of illness anxiety in patients with SSD, as meta-analysis revealed higher illness anxiety in patients with SSD compared with healthy controls and even compared with patients with illness anxiety disorder.^17^ While SSD is characterized by distressing somatic symptoms accompanied by excessive thoughts, feelings, or behaviors, such as symptom-related anxiety, illness anxiety disorder (IAD) is defined by excessive health-related worry in the absence of significant somatic symptoms.^1^ Conceptually, health anxiety can be considered a central transdiagnostic mechanism that operates across both SSD and IAD, influencing symptom severity, functional impairment, and treatment needs. Due to this conceptual overlap, the authors argue that illness anxiety should not be considered a distinct psychological risk factor for SSD.^17^ Given the clinical relevance of the association between SSD severity and functional impairment as demonstrated in several studies,^5^ future research should focus on longitudinal data to identify predictors of SSD severity and clarify the role of somatic comorbidity in this regard.

### Strengths and Limitations

An important strength of the present study is the systematic use of the Structured Clinical Interview for DSM-5 (SCID-5) for diagnosing SSD. While the SCID-5 is considered the gold standard for SSD diagnosis, the application involves a degree of subjectivity, partly due to somewhat vague criteria, such as the phrase “excessive thoughts, feelings, and behaviors,” which may be interpreted differently by individual raters. Consequently, researchers have called for further specification and operationalization of the SSD diagnostic criteria.^5^ Another strength is the systematic and elaborate assessment of somatic comorbidity by a trained clinician using the CIRS, which allows for a structured evaluation across 13 organ systems.^35^ This method improves diagnostic objectivity and accuracy compared with self-report and has demonstrated good interrater reliability,^55^ supporting consistency across raters. However, the use of the CIRS is not totally free of limitations, as the 13 organ system categories are not fully comparable and may differ in their potential impact on the overall health (eg, impairment in the cardiac system vs. in the ear, nose, throat, or eye). To address this issue, Miller et al^35^ have proposed weighting factors to account for differences in disease severity. In the present study, participants were categorized based on the CIRS comorbidity index into 2 groups: those with somatic comorbidity (index ≥1) and those without (index=0). This binary classification focuses solely on the presence or absence of somatic comorbidity and does not account for multimorbidity or the severity of individual conditions. Furthermore, mild impairments across multiple systems—although clinically relevant—are not reflected by the comorbidity index. The CIRS scoring system does not allow for a detailed assessment of specific diagnoses, limiting insight into the role of particular diseases. Only low to moderate levels of somatic comorbidity were found in the present study sample, as indicated by a mean CIRS total score of 4 out of a possible 52. This may be partially due to the recruitment of participants from a psychosomatic outpatient setting, where participants were limited to those able to attend outpatient care. In addition, the single-center design from a tertiary care setting may have resulted in a selective sample that is not fully representative of the broader population of individuals with SSD. Consequently, the generalizability of our findings to populations with more severe somatic illnesses, from other clinical settings or from the general population remains to be further investigated.

Another limitation is that psychiatric diagnoses were not assessed in the present analysis. Psychiatric comorbidity is likely to influence several of the parameters examined in this study (eg, psychological burden, quality of life and health care utilization). Consequently, the results regarding differences and similarities between patients with and without somatic comorbidity should be interpreted with caution, as variables such as psychiatric comorbidities may partially account for variability in SSD severity and psychosocial outcomes.

Finally, causal relationships between SSD severity and associated variables were not analyzed due to the cross-sectional design of the present study. However, longitudinal data will be available for future analyses as part of the SOMA.SSD project.^10^

## CONCLUSION

The present study offers an in-depth characterization of somatic comorbidity in patients with Somatic Symptom Disorder (SSD) and provides evidence that the clinical presentation and severity of SSD do not differ substantially between patients with and without somatic comorbidity in a psychosomatic outpatient setting. Somatic comorbidities are common in patients with SSD. Our findings suggest that, cross-sectionally, they do not lead to large differences in SSD severity or psychosocial profile. However, smaller effects or specific mechanisms related to comorbid physical illness cannot be ruled out and warrant further investigation in future studies. Although patients with somatic comorbidity were older and reported lower physical quality—but higher mental—health-related quality of life, there were no significant differences between groups in terms of psychological symptom burden, HCU, or SSD severity. Illness anxiety emerged as the only variable significantly associated with SSD severity. These findings support the validity and applicability of the SSD diagnosis regardless of the presence of somatic comorbidity. Future research should focus on the impact of somatic disease severity and multimorbidity in more diverse clinical populations and investigate the role of somatic comorbidity in longitudinal designs.

*Source of Funding and Conflicts of Interest: This project is part of the Research Unit 5211 (FOR 5211) Persistent SOMAtic Symptoms ACROSS Diseases: From Risk Factors to Modification (SOMACROSS) and has received funding from the German Research Foundation (Deutsche Forschungsgemeinschaft, DFG; grant numbers NE 1635/3-1 and TO 908/2-1). The authors report no conflicts of interest.*

*Transparency and Openness Promotion: The study design and analysis plan were preregistered at http://www.isrctn.com/ISRCTN36251388. Detailed information on study procedures, sample size determination, and measures is provided in the study protocol.7 Study data and analysis code are available upon reasonable request from the corresponding author.*

## Supplementary Material
