# Supplementary material for: Psychosocial and Clinical Correlates of Somatic Symptom Disorder in Patients With and Without Somatic Comorbidities: Cross-Sectional Findings From the SOMA.SSD Study
Source: Biopsychosoc Sci Med. 2026 Apr 14;88(5):464–74. doi: 10.1097/PSY.0000000000001483 (PMC13220931; doi:10.1097/PSY.0000000000001483)
Supplement: Supplementary file 1 [file psy-88-464-s001.docx]

Figure S1: Study Flow

**Assessed for eligibility**

n = 1594

**Excluded at screening**

not reachable, not interested, too busy, too ill, awaiting immediate treatment

n = 412

**Fulfilled SSD screening criteria**

**(PHQ-15 ≥ 5 and SSD-12 ≥ 20)**

n = 769

**SCID-5 interviews conducted**

n = 357

**No SSD diagnosis by SCID-5**

n = 86

**Excluded prior to participation**

not reachable, not interested anymore, too busy

n = 30

Note: SSD = Somatic Symptom Disorder; SCID = Structured Clinical Interview for DSM-5; PHQ-15 = Patient Health Questionnaire-15; SSD-12 = Somatic Symptom Disorder ̶ B-criteria Scale

**Study sample**

n = 241

**SSD diagnosis confirmed by SCID-5**

n = 271
