# Supplementary material for: Psychosocial and Clinical Correlates of Somatic Symptom Disorder in Patients With and Without Somatic Comorbidities: Cross-Sectional Findings From the SOMA.SSD Study
Source: Biopsychosoc Sci Med. 2026 Apr 14;88(5):464–74. doi: 10.1097/PSY.0000000000001483 (PMC13220931; doi:10.1097/PSY.0000000000001483)
Supplement: Supplementary file 2 [file psy-88-464-s002.docx]

**Table S1: Correlation of clinical variables with somatic comorbidity (CIRS total score) (n = 241)**

| **Variable** | **n** | ***r*** | ***p* -value** | |
| --- | --- | --- | --- | --- |
| **Health-related quality of life (SF-12)** |  |  |  | |
| Physical component | 239 | -0.351 | **<.001** | |
| Mental component | 239 | 0.123 | .057 | |
| **Somatic comorbidity self-report (SCQ-D)** | 241 | 0.611 | **<.001** | |
| **Somatic symptom burden (PHQ-15)** | 240 | 0.214 | **<.001** | |
| **Somatic symptom disorder - B-criteria scale (SSD-12)** | 238 | 0.179 | **.006** | |
| **Depression severity (PHQ-9)** | 240 | 0.128 | **.048** | |
| **Anxiety severity (GAD-7)** | 240 | 0.065 | .316 | |
| **Illness anxiety (WI-7)** | 239 | 0.202 | **.002** | |
| **Perceived stress (PSS-10)** | 239 | 0.078 | .231 | |
| **Somatosensory amplification (SSAS)** | 239 | 0.132 | **.042** | |
| Note: CIRS = Cumulative Illness Rating Scale; SSD = Somatic Symptom Disorder; SCQ-D = Selfadministered Comorbidity Questionnaire (depression excluded); PHQ-15 = Patient Health Questionnaire-15; SSD-12 = Somatic Symptom Disorder B-criteria Scale; SF-12 = Short Form Health Survey; PHQ-9 = Patient Health Questionnaire-9; WI-7 = Whiteley Index-7; SSAS = Somatosensory Amplification Scale; GAD-7 = Generalized Anxiety Disorder Scale-7; PSS-10 = Perceived Stress Scale-10. | | | |  |
